# Iron from nanostructured ferric phosphate: absorption and biodistribution in mice and bioavailability in iron deficient anemic women

**DOI:** 10.1038/s41598-022-06701-x

**Published:** 2022-02-18

**Authors:** Jeannine Baumgartner, Hans Christian Winkler, Lizelle Zandberg, Siriporn Tuntipopipat, Phatchari Mankong, Cor Bester, Florentine Hilty, Jan Rijn Zeevaart, Sueppong Gowachirapant, Michael B. Zimmermann

**Affiliations:** 1 Laboratory of Human Nutrition, Department of Health Sciences and Technology, ETH Zurich, Schmelzbergstrasse 7, 8092 Zurich, Switzerland; 2 Center of Excellence in Nutrition, North-West University, Potchefstroom, South Africa; 3 Institute of Nutrition, Mahidol University, Salaya, Nakhon Pathom, 73170 Thailand; 4 DST/NWU Preclinical Drug Development Platform, North-West University, Potchefstroom, South Africa; 5 South African Nuclear Energy Corporation South Africa (Necsa), Pelindaba, South Africa

**Keywords:** Nanobiotechnology, Haematological diseases

## Abstract

Food fortification with iron nanoparticles (NPs) could help prevent iron deficiency anemia, but the absorption pathway and biodistribution of iron-NPs and their bioavailability in humans is unclear. Dietary non-heme iron is physiologically absorbed via the divalent metal transporter-1 (DMT1) pathway. Using radio- iron isotope labelling in mice with a partial knockdown of intestine-specific DMT1, we assessed oral absorption and tissue biodistribution of nanostructured ferric phosphate (FePO_4_-NP; specific surface area [SSA] 98 m^2^g^-1^) compared to to ferrous sulfate (FeSO_4_), the reference compound. We show that absorption of iron from FePO_4_-NP appears to be largely DMT1 dependent and that its biodistribution after absorption is similar to that from FeSO_4_, without abnormal deposition of iron in the reticuloendothelial system. Furthermore, we demonstrate high bioavailability from iron NPs in iron deficient anemic women in a randomized, cross-over study using stable-isotope labelling: absorption and subsequent erythrocyte iron utilization from two ^57^Fe-labeled FePO_4_-NP with SSAs of 98 m^2^g^−1^ and 188 m^2^g^−1^ was 2.8-fold and 5.4-fold higher than from bulk FePO_4_ with an SSA of 25 m^2^g^−1^ (*P* < 0.001) when added to a rice and vegetable meal consumed by iron deficient anemic women. The FePO_4_-NP 188 m^2^g^-1^ achieved 72% relative bioavailability compared to FeSO_4_. These data suggest FePO_4_-NPs may be useful for nutritional applications.

## Introduction

Iron deficiency anemia (IDA) is a major global health burden and women of childbearing age are particularly vulnerable^1^. Iron fortification of foods is a recommended strategy to prevent IDA, but iron fortification remains a challenge, because well absorbed, water-soluble iron compounds, e.g. ferrous sulfate (FeSO_4_), often cause unacceptable sensory changes when added to foods^2^. Poorly acid-soluble iron compounds, such as ferric phosphate (FePO_4_) in bulk form, are stable in foods but their absorption in humans is too low to have nutritional value^3^. However, the dissolution and absorption of poorly-soluble iron compounds is inversely related to particle size and nanostructured iron compounds may be useful as food fortificants or supplements^4^. We have previously shown that scalable nanostructured FePO_4_ (FePO_4_-NP) produced by flame spray pyrolysis (FSP) dissolve rapidly in gastric conditions^5^. In an anemic rat model, iron bioavailability (determined as the ability to rapidly replete hemoglobin (Hb) when given orally) from two FePO_4_-NP compounds (FePO_4_/Fe_2_O_3_, specific surface area [SSA] 197 m^2^/g and FePO_4_/Zn_3_(PO_4_)_2_, SSA 191 m^2^/g) was 75 and 95%, respectively, compared to the ionic reference compound FeSO_4_^5^. However, rodents absorb iron efficiently because they endogenously synthesize ascorbic acid, have lower duodenal pH, and are less affected by dietary absorption inhibitors than humans^6,7^. Thus, whether the high bioavailability of FePO_4_-NP determined in rats can be extrapolated to humans is unknown, since existing animal models were never validated with nano compounds.

Moreover, the mechanism of uptake of iron NPs from the gastrointestinal tract is uncertain. Ferrous iron from fortificants and foods is absorbed in the duodenum via the divalent metal transporter 1 (DMT1)^2,8^. Ferric iron must be reduced to the ferrous state before uptake by DMT1^9,10^. Since iron cannot be actively excreted in mammals and iron overload is toxic, iron absorption through DMT1 is tightly regulated by iron stores and circulating hepcidin^11^. Because of their small size, iron NPs could be absorbed through other pathways^12,13^, and unregulated translocation from the gut into body tissues might be toxic. In Caco-2 cell models, endocytosis of iron NPs has been described^13^, but siRNA-mediated knockdown of DMT1 reduced iron uptake from FePO_4_-NP in Hutu-80 cell models by 50%^14^. Whether iron from FePO_4_-NP can be absorbed independently of the DMT1 pathway in vivo remains uncertain.

Therefore, our study objectives were: (1) in mouse models, (i) to compare Hb trajectories (iron absorption and utilization) in intestine-specific DMT1 partial knockdown (DMT1^int/int^) mice and homozygously floxed (DMT1^fl/fl^) controls fed diets containing FePO_4_-NP (SSA 98 m^2^g^−1^) or FeSO_4_ (positive reference compound) for 18 days; and (ii) to compare absorption and biodistribution of iron from a single oral dose of FePO_4_-NP (SSA 98 m^2^g^−1^) and FeSO_4_ labelled with radioactive iron (^59^Fe) in iron deficient anemic DMT1^int/int^ and DMT1^fl/fl^ control mice; (2) in iron deficient anemic women, to measure iron absorption and erythrocyte iron utilization (bioavailability) from a rice and vegetable meal fortified with large and small FePO_4_-NP (SSA 98 m^2^ g^−1^ and 188 m^2^ g^−1^) labeled with a stable iron isotope (^57^Fe), compared to ^58^Fe-labeled bulk FePO_4_ and FeSO_4_ as negative and positive reference compounds, respectively.

## Results

### Iron nanoparticle production and characterization

**Figure 1:**
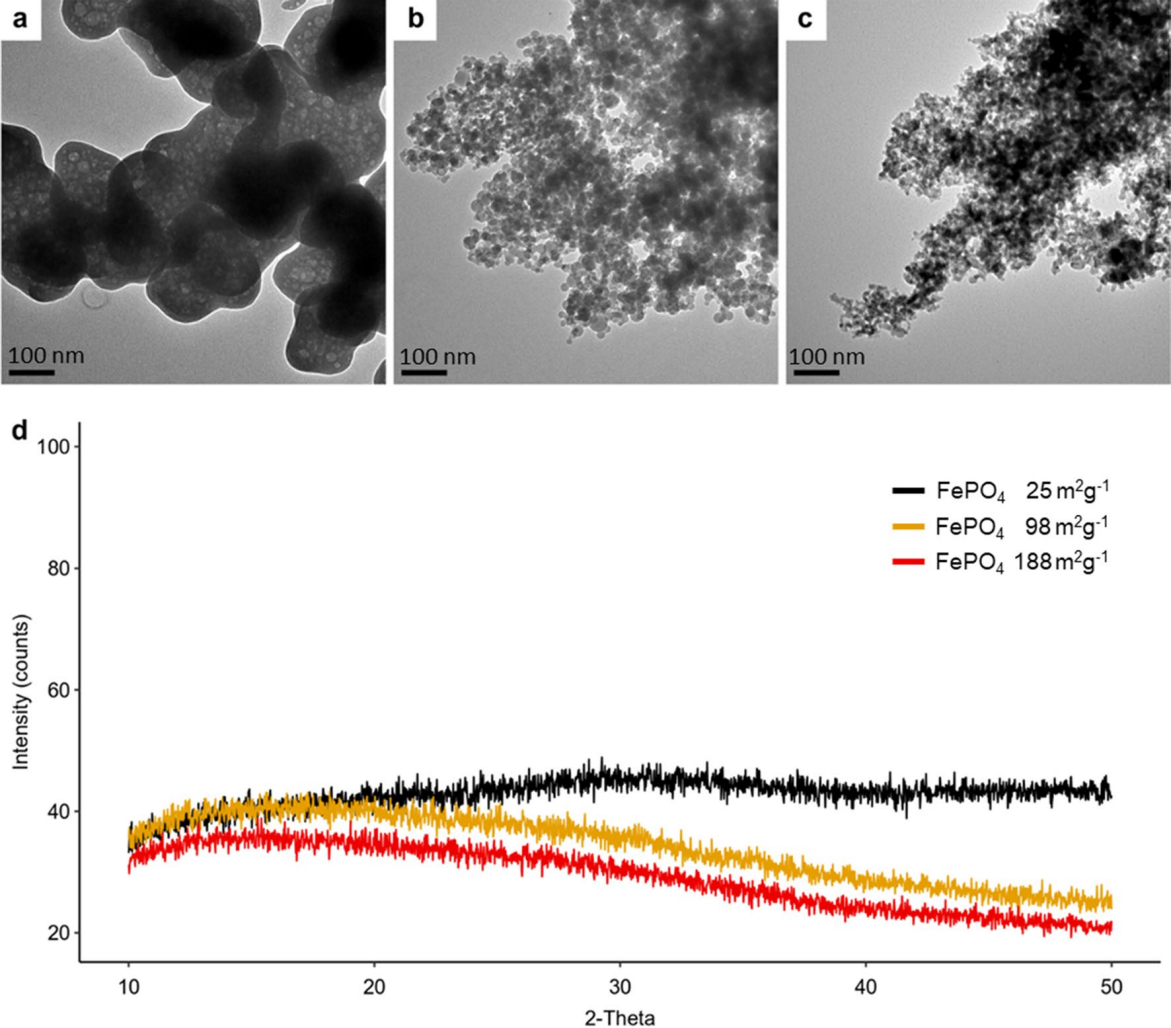
Particle characterization. **(a–c)** TEM micrographs of **(a)** bulk FePO_4_, **(b)** 98 m^2^ g^−1^ FePO_4_ and **(c)** 188 m^2^ g^−1^ FePO_4_ , scale bars: 100 nm. (**d)** XRD patterns of 25 m^2^ g^−1^, 98 m^2^ g^−1^ and 188 m^2^ g^−1^ FePO_4_. The absence of peaks in the X-ray diffraction (XRD) analysis indicates that all compounds were XRD amorphous. X-axis indicates the diffraction angle (2-Theta), Y-axis indicates the counts.

Two different sized ferric orthophosphate dihydrate nanoparticles (FePO_4_-NPs) with SSAs of 188 m^2^ g^−1^ and 98 m^2^ g^−1^ were produced by flame spray pyrolysis (FSP) at ETH Zurich as previously described^15^ with adaptations (see “Methods”). FSP is a scalable production process^16^ for tailor-made particles with high SSA and well-defined chemical composition^17^. As indicated by transmission electron microscopy (TEM) images and X-ray diffraction (XRD), the two FePO_4_-NPs are almost spherical particles with a calculated size (dBET) of 11 nm and 21 nm, respectively (Fig. 1a-c) and XRD-amorphous (Fig. 1d) . Amorphous ferric orthophosphate with a SSA of 25 m^2^ g^−1^ served as reference compound for BET and XRD measurements. FePO_4_-NPs for the human study were produced from stable isotope (^57^Fe) enriched precursors, bulk FePO_4_ and FeSO_4_ from (^58^Fe) enriched precursors. For the human study, bulk size FePO_4_ with a SSA of 27 m^2^ g^−1^ was produced as previously described^18^ with adaptations (see “Methods”). As indicated by TEM, these particles have a size above 100 nm and an irregular shape. The calculated primary particle size of these FePO_4_ particles with an SSA of 27 m^2^ g^−1^ would be 77 nm; however, as shown in the TEM images, the assumption of sphericity is not valid and we therefore refer to these particles as bulk FePO_4_. For the methods of SSA, hydrodynamic diameter, XRD and TEM, see “Methods”. In ultrapure water, bulk FePO_4_ and the 98 m^2^ g^−1^ and 188 m^2^ g^−1^ FePO_4_-NPs formed large agglomerates with a mean diameter and polydispersity index (PDI) of 859 ± 152 nm (PDI 0.63) at pH 6.7, 1191 ± 603 nm (PDI 0.34) at pH 6.1 and 1149 ± 132 nm (PDI 0.41) at pH 6.0 and a Zeta potential of –5.3 ± 1.6 mV, −2.1 ± 0.1 mV and -2.5 ± 0.1 mV. The same FePO_4_-NPs were shown to form stable agglomerates for up to 72 h in aqueous dispersions with added protein content (10% FCS) with a mean diameter of 231–615 nm^19^. Particle characterization followed MIRIBEL guidelines^20^.

### Characterization of intestine-specific DMT1 knockdown model

**Figure 2:**
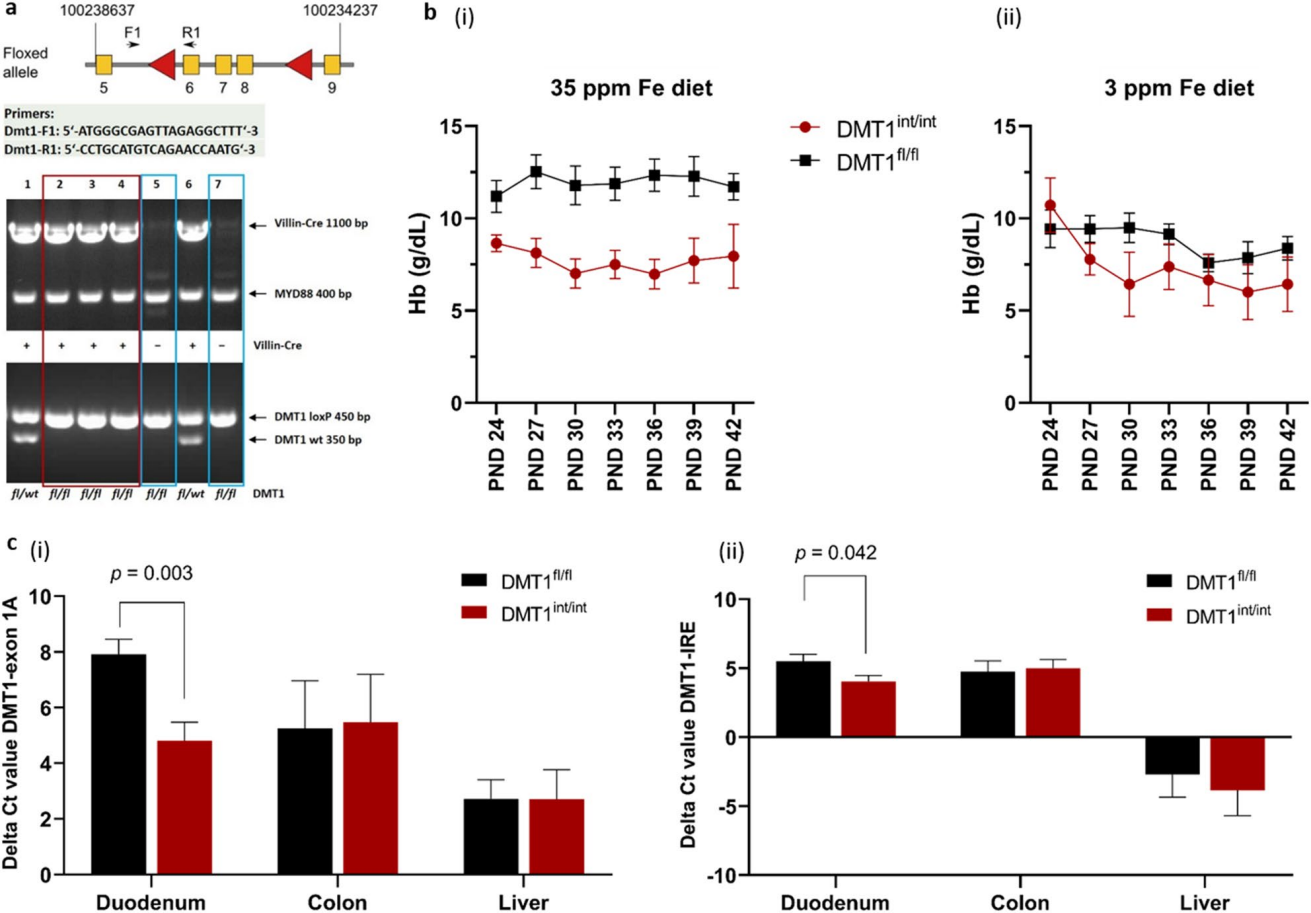
Characterization of the DMT1^int/int^ mouse model. **(a)** Schematic illustration of the floxed allele and primer positions used to confirm excision by PCR genotyping of intestine knockdown (DMT1^int/int^, lane 2–4, red box) and control (DMT1^fl/fl^, lane 5 and 7, blue boxes) mice. Image was cropped and compiled from original images available as Supplementary Figs. 1 and 2. (**b)** Haemoglobin (Hb) trajectory (PND 24‒42) in DMT1^int/int^ (n = 6 male; n = 4 female) and DMT1^fl/fl^ (n = 10 male; n = 11 female) mice receiving the AIN93G diet containing (i) 35 ppm iron (as ferrous citrate) and (ii) 3 ppm iron (native). Results are shown as means ± SEM. **c,** Expression of (i) DMT1-exon1A and (ii) DMT1-IRE mRNA in duodenum, colon and liver of 42-day-old DMT1^int/int^ and DMT1^fl/fl^ mice fed an iron deficient (3 ppm iron) diet from PND 24‒42, normalized to 18S and βActin as endogenous reference genes to calculate delta Ct values. Differences in gene expression by genotype (DMT1^int/int^ vs. DMT1^fl/fl^) were determined by two-sided independent t-tests. The results are shown as means ± SEM and differences were considered significant at p < 0.05.

We chose DMT1^int/int^ and DMT1^fl/fl^ littermates (controls) as the model to study absorption and biodistribution of nanosized FePO_4_ (SSA 98 m^2^ g^−1^). DMT1^int/int^ and DMT1^fl/fl^ mice were bred on a C57BL/6 background by crossing floxed DMT1 (courtesy of Nancy Andrews, Duke University, USA^21^) with villin-Cre transgenic mice. Figure 2a shows a schematic illustration of the floxed allele and the primer positions used for genotyping of the DMT1^int/int^ and DMT1^fl/fl^ mice. Homozygously floxed DMT1^fl/fl^ mice that do not carry the Cre recombinase were chosen as controls in all mice experiments to account for a potential effect of floxing on iron absorption and biodistribution. For details of breeding, genotyping and housing see “Methods”.

To validate the model and to determine the optimal age window for the feeding and radio-isotope experiments, we assessed Hb concentration trajectories of DMT1^int/int^ and DMT^fl/fl^ mice fed an iron-sufficient (35 ppm iron as ferrous citrate) or iron deficient (3 ppm native iron) diet from postnatal day (PND) 24 (weaning) until 42. PND 42 was set as endpoint, as it was the age when first mice reached a Hb < 4 g/dL, which we defined as the humane endpoint (see “Methods” for definition)^22^. We measured Hb in tail blood spots at PND 24, 27, 30, 33, 36, 39, and 42. Using repeated-measures ANOVA with time as within-group factor, and dietary iron content (35 ppm vs. 3 ppm) and genotype (DMT1^int/int^ vs. DMT^fl/fl^) as between-group factors, we found a significant effect of iron content (p = 0.023) for lower Hb in mice receiving the 3 ppm iron diet, and of genotype (p = 0.006) for lower Hb in DMT1^int/int^ mice (Fig. 2b). There was no significant iron content x genotype interaction, indicating that both the 3 ppm iron diet and the intestine-specific DMT1 knockdown independently lowered Hb across PND 24–42. However, we obtained no significant time x iron content (p = 0.087) or time x genotype (p = 0.090) interactions, indicating that the Hb-lowering effect of the 3 ppm diet and the intestine-specific DMT1 knockdown was not time-dependent during this age period. These results confirm that the DMT1^int/int^ mice bred for our studies have significantly lower Hb concentrations from PND 24–42, both when fed an iron-sufficient or iron deficient diet, but there was no progressive decline in Hb concentrations, suggesting upregulation of iron absorption from a partly intact DMT1 pathway.

We further determined expression of DMT1 mRNA harboring an iron-responsive element (IRE) in its 3’-terminal exon and the upstream 5’ exon1A in crude homogenates prepared from whole duodenum (not isolated enterocytes), colon and liver of DMT^int/int^ and DMT^fl/fl^ mice fed an iron deficient diet from PND 24‒42 (Fig. 2c). The DMT1-IRE and DMT1-exon1A isoforms were previously implicated in iron regulation, specifically in the duodenum^23^. DMT1 mRNA levels are expected to be increased in the duodenum of iron deficient mice with a functional DMT1 gene^23^. We showed that expression of both exon1A and the IRE of DMT1 was significantly lower in the duodenum of DMT1^int/int^ compared to DMT1^fl/fl^ mice fed an iron deficient diet. However, the reduction in DMT1 mRNA expression was partial, suggesting incomplete knockdown of DMT1 in the DMT1^int/int^ mice. This is supported by the observation that expression of the DMT1-IRE isoform in the duodenum was positively associated with Hb concentrations measured at PND42 in DMT1^fl/fl^ mice receiving 3 ppm and 35 ppm iron (standardized β = 0.623, p = 0.026; regression models controlled by sex), with the same trend observed in DMT1^int/int^ mice (standardized β = 0.522, p = 0.079) (data not shown). There were no differences in expression of DMT1 mRNA between DMT1^int/int^ and DMT1^fl/fl^ mice in colonic and liver tissue, confirming the intestine-specificity of the partial knockdown.

### Eighteen-day FePO_4_-NP feeding study in DMT1^int/int^ and DMT1^fl/fl^ mice

**Figure 3:**
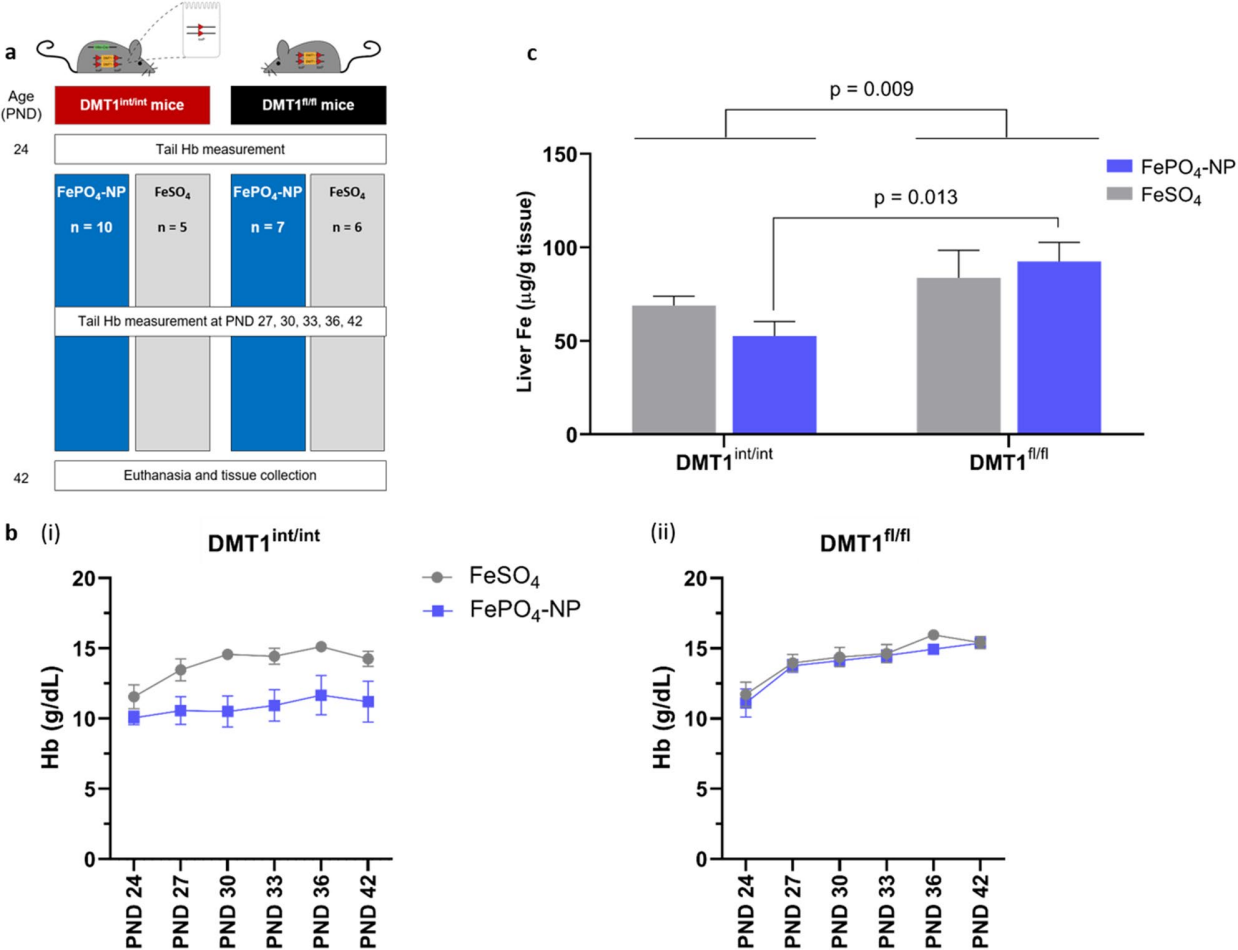
Eighteen-day feeding study in DMT1^int/int^ and DMT1^fl/fl^ mice. **(a)** Outline of feeding study. (**b)** Hemoglobin (Hb) trajectory in (i) DMT1^int/int^ (n = 6 male; n = 9 female) and (ii) DMT1^fl/fl^ (n = 6 male; n = 7 female) mice receiving the AIN93G diet fortified with FePO_4_-NP (SSA 98 m^2^ g^−1^) or FeSO_4_ (reference compound). Results are shown as means ± SEM. (**c)** Liver iron concentrations in DMT1^int/int^ and DMT1^fl/fl^ mice fed diets fortified with 35 ppm FeSO_4_ or FePO_4_-NP for 18 days (postnatal days [PND] 24‒42). Differences in liver iron concentrations by genotype (DMT1^int/int^ vs. DMT1^fl/fl^) and by iron compound (FePO_4_-NP vs. FeSO_4_) were determined by two-sided independent t-tests. The results are shown as means ± SEM and differences were considered significant at p < 0.05.

To determine whether FePO_4_-NP is absorbed via DMT1 and utilized for erythropoiesis, we compared Hb trajectories between DMT1^int/int^ and DMT1^fl/fl^ (control) mice fed with FePO_4_-NP (SSA 98 m^2^ g^−1^) or FeSO_4_ as the reference compound from PND 24–42 (18 days). We hypothesized that (1) intestine-specific DMT1 knockdown would lower Hb equally in mice fed FePO_4_-NP or FeSO_4_ and, (2) that Hb would be comparable in DMT1^fl/fl^ mice fed iron in the form of nanosized FePO_4_-NP or FeSO_4_. We randomly allocated a total of 28 male and female DMT1^int/int^ and DMT1^fl/fl^ mice to receive either the purified AIN-93G diet containing 35 ppm iron as FePO_4_-NP or FeSO_4_ from PND 24–42. The design of the study is shown in Fig. 3a. For more details see “Methods”. We measured Hb in tail blood spots at PND 24, 27, 30, 33, 36 and 42. Using repeated-measures ANOVA with age as within-group factor, and iron compound (FePO_4_-NP vs. FeSO_4_) and genotype (DMT1^int/int^ vs. DMT^fl/fl^) as between-group factors, we found a significant effect of genotype (p = 0.042) for lower Hb concentrations in DMT1^int/int^ mice, and a nonsignificant effect of iron compound (p = 0.067) for lower Hb in mice fed FePO_4_-NP (Fig. 3b). Although the interaction between genotype and iron compound was not significant (p = 0.140), the Hb-lowering effect of the DMT1 knockdown appeared to be compound-specific, in that Hb trajectories were similar between compounds in DMT1^fl/fl^ mice, but Hb concentrations were lower in DMT1^int/int^ mice fed FePO_4_-NP than fed FeSO_4_. The data in Fig. 3b suggest the FeSO_4_ was nearly as effective at increasing Hb in DMT1^int/int^ mice as it was in DMT1^fl/fl^ mice, whereas for FePO_4_-NP, Hb concentrations were increased in the DMT1^fl/fl^ mice but were unchanged in the DMT1^int/int^ mice. This pattern is supported by the liver iron concentrations (Fig. 3c) at PND 42 in DMT1^int/int^ and DMT1^fl/fl^ mice fed FePO_4_-NP or FeSO_4_. We found a significant effect of genotype (p = 0.009) for lower liver iron concentrations in DMT1^int/int^ mice. Specifically, FePO_4_-NP feeding resulted in significantly lower liver iron concentrations in DMT1^int/int^ compared to DMT1^fl/fl^ mice, but FeSO_4_ did not. The lacking effect of the intestinal DMT1 knockdown on Hb trajectories and liver iron concentrations in mice fed a diet containing iron as FeO_4_-NP may be explained by the high solubility of FeSO_4_ at low gastric pH and rodents’ high efficiency in absorbing iron^6,7^. Considering that our model was not a complete intestinal DMT1 knockout model (described above), it can be speculated that rapid dissolution in the proximal gut and absorption of FeSO_4_ in mice with a partial intestinal DMT1 knockdown was enough to maintain erythropoiesis and liver iron stores during the experimental period (PND 24–42). This may also explain why the DMT^int/int^ mice fed (and bred on) ferrous citrate in the characterization experiment were more anemic from PND 24–42 than the DMT^int/int^ mice fed (and bred on) FeSO_4_ in the feeding experiment. Taken together, these data suggest that compared to FeSO_4_, FePO_4_-NP were less well absorbed in DMT1^int/int^ compared to DMT1^fl/fl^ mice, suggesting that absorption of iron from FePO_4_-NP is more sensitive to partial knockdown of DMT1.

### Absorption and biodistribution of radiolabeled FePO_4_-NP and FeSO_4_ from a single oral dose in iron deficient anemic DMT1^int/int^ and DMT1^fl/fl^ mice

**Figure 4:**
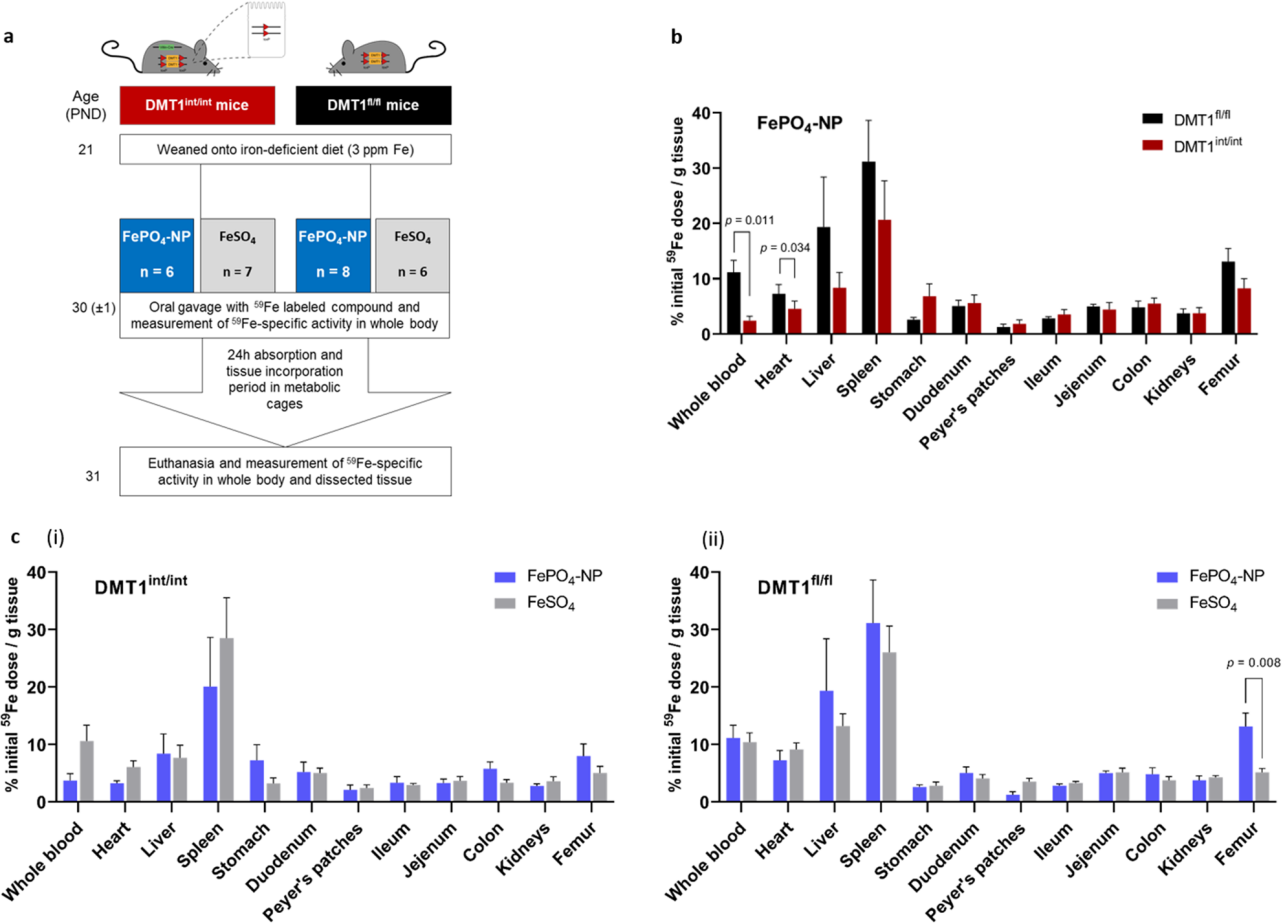
Absorption and biodistribution of a single oral dose of radiolabeled FePO_4_-NP and FeSO_4_ iron deficient anemic DMT1^int/int^ and DMT1^fl/fl^ mice. **(a**) Outline of radioisotope study. (**b**) Biodistribution of ^59^Fe (% of initial ^59^Fe/g tissue) from a single dose (~ 50 µg) of FePO_4_-NP (SSA 98 m^2^ g^−1^) after oral gavage in iron deficient anemic DMT1^fl/fl^ (n = 7 male; n = 5 female) and DMT1^int/int^ (n = 7 male, n = 8 female) mice. Differences in tissue ^59^Fe distribution by genotype (DMT1^int/int^ vs. DMT1^fl/fl^) were determined by two-sided independent t-tests. The results are shown as means ± SEM and differences were considered significant at p < 0.05. (**c**) Biodistribution of ^59^Fe from FePO_4_-NP and FeSO_4_ (reference compound) 24 h after oral gavage in iron deficient anemic (i) DMT1^int/int^ mice and (ii) DMT1^fl/fl^ mice. Differences in tissue ^59^Fe distribution by iron compound (FePO_4_-NT vs. FeSO_4_) were determined by two-sided independent t-tests. The results are shown as means ± SEM and differences were considered significant at p < 0.05.

To determine whether absorption of FePO_4_-NP is DMT1-dependent and biodistribution comparable to FeSO_4_ in iron deficient anemic conditions, we administered a single oral dose of irradiated FePO_4_-NP (SSA 98 m^2^g^−1^) or FeSO_4_ labelled with radioactive iron (^59^Fe) to iron deficient anemic DMT1^int/int^ and DMT1^fl/fl^ control mice and measured tissue distribution of ^59^Fe 24 h after administration. Based on the findings from the feeding study, we hypothesized that less iron from a single oral dose of FePO_4_-NP would be absorbed in DMT1^int/int^ than DMT1^fl/fl^ mice. We further hypothesized that tissue distribution of iron taken up and absorbed from the single oral dose of FePO_4_-NP would be equal to FeSO_4_ in both iron deficient anemic DMT1^int/int^ and DMT1^fl/fl^ control mice. A total of 27 male and female DMT1^int/int^ and DMT1^fl/fl^ mice were randomly allocated by genotype to receive radiolabeled FePO_4_-NP (SSA 98 m^2^g^-1^) or FeSO_4_ and were placed on an iron deficient diet (3 ppm native iron) from PND 21 until the end of the experiment. At PND 24, mice were transported to the Nuclear Energy Corporation South Africa (NECSA) and acclimatized until administration of radiolabeled FePO_4_-NP or FeSO_4_ by oral gavage at PND 30. The design of the study is shown in Fig. 4a. The day before compound administration, Hb was measured in a tail blood spot, and 2 h before administration mice were fasted. A single dose of ~ 50 µg iron (mean ± SEM administered: 47.4 ± 2.9 µg) in the form of the allocated compound labelled with ^59^Fe (FePO_4_-NP: 0.08 ± 0.01 MBq; FeSO_4_: 0.52 ± 0.2 MBq) was orally gavaged (in 100 µL saline containing 0.1% bovine serum albumin) and total dose administered determined by measuring syringe activity before and after gavage using a dose calibrator (Capintec CRC-15R, Capintec Inc., Ramsey, NJ, USA). Mice were then placed into a clean metabolic cage and received ad libitum access to iron deficient diet (3 ppm iron) one hour after oral gavage. After 24 h, mice were euthanized and individual tissues were dissected and analyzed for ^59^Fe content using a gamma-counter. All counts were adjusted for decay. For more details see “Methods”.

DMT1^int/int^ mice had significantly lower Hb than DMT1^fl/fl^ mice (5.7 ± 0.5 vs. 8.9 ± 0.7 g/dL, p = 0.01) the day before compound administration. As shown in Fig. 4b, 24 h after administration, significantly less ^59^Fe from FePO_4_-NP was present in whole blood and heart (expressed as % initial ^59^Fe dose/g tissue) in DMT1^int/int^ compared to DMT^fl/fl^ mice. These data are consistent with lower absorption of FePO_4_-NP in DMT1^int/int^ compared to DMT^fl/fl^ mice shown in the feeding study above (Fig. 3b,c). Notably, in DMT1^int/int^ and DMT1^fl/fl^ mice (Fig. 4c), we found no significant difference in tissue ^59^Fe distribution between FePO_4_-NP and FeSO_4_. The only exception was in DMT1^fl/fl^ mice, where significantly more ^59^Fe from FePO_4_-NP was present in femoral bone marrow than from FeSO_4_ (Fig. 4c,ii). Overall, the results from this study indicate that in iron deficient anemic mice, iron from a single oral dose of FePO_4_-NP displays a similar biodistribution as FeSO_4_.

### Iron bioavailability of two ^57^Fe-labeled FePO_4_-NP in iron deficient anemic women

To quantify iron absorption and erythrocyte incorporation (bioavailability) from two FePO_4_-NPs (SSA of 98 m^2^ g^−1^ and 188 m^2^ g^−1^) in iron deficient, mostly anemic women, we fortified two standardized rice test meals with FePO_4_-NPs labeled with stable iron isotopes and compared their performance to labeled bulk FePO_4_ with a SSA of 25 m^2^g^−1^ (as the negative reference compound) and dried FeSO_4_ (as the positive reference compound) in a randomized, cross-over study. We hypothesized that: (1) an increase in SSA of the FePO_4_-NP compound would significantly increase iron bioavailability, and (2) iron bioavailability from FePO_4_-NP SSA 188m^2^ g^−1^ would not differ significantly compared to iron bioavailability from FeSO_4_. Iron bioavailability was estimated by using stable-isotope techniques measuring the incorporation of ^57^Fe and ^58^Fe into erythrocytes 14 days after administration^24^.

**Figure 5:**
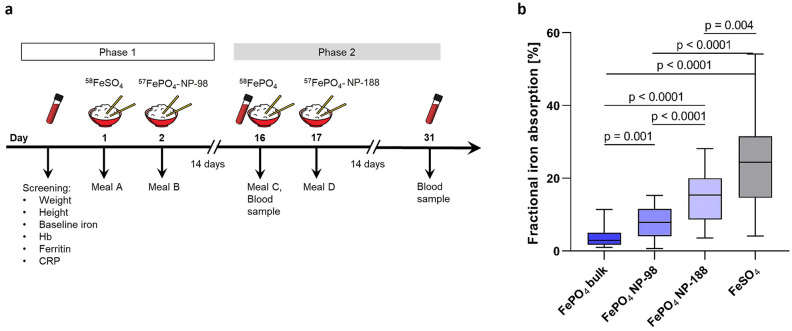
Iron bioavailability of two ^57^Fe-labeled FePO_4_-NP in iron deficient anemic women (n = 18). **(a)** Outline of randomized cross-over study. (**b)** Fractional iron absorption (%) from rice test meals fortified with FePO_4_-NPs (SSA 98 m^2^g^−1^ and 188 m^2^ g^−1^) labeled with a stable isotope (^57^Fe), and ^58^Fe-labeled bulk FePO_4_ (SSA 25 m^2^ g^−1^) and FeSO_4_ as negative and positive reference compounds, respectively. Meal sequence was randomized across all women. Pairwise comparisons were performed using two-sided paired t-tests with Bonferroni adjustment for multiple testing. Boxes indicate median and interquartile ranges, whiskers describe the range of the data (min to max). Differences were considered significant at p < 0.05. *CRP* C-reactive protein, *Hb* hemoglobin.

The study was carried out in 18 Thai women who provided informed written informed consent. Main inclusion criteria were: (1) female aged 18 to 49 years and (2) Hb ≥ 80 g/L and plasma ferritin < 25 µg/L (see “Methods” for additional inclusion criteria and details on methods). We chose Thailand as our study site because, worldwide, the greatest number of women with anemia live in the WHO South and Southeast Asia Region, and in Thailand, 32% of women of reproductive age are anemic^25^. The study had a cross-over design in which all women consumed four labeled test meals over a 31-day period (Fig. 5a). The order of the four test meals was randomly assigned to each subject. In a first phase, the women presented fasting and consumed the first test meal between 7.00 and 9.00 am on day 1 and the second test meal between 7.00 and 9.00 am on day 2; in the second phase, 14 days later, they consumed the remaining 2 test meals between 7.00 and 9.00 am on days 16 and 17. Consumption of meals was directly supervised and all women completed all meals. No intake of food and fluids was allowed for 2 h afterward. The standardized test meal consisted of rice with vegetables (see “Methods”) fortified with 2 mg of isotopically labeled compound and a glass of deionized water (200 ml). The two FePO_4_-NPs were labeled with ^57^Fe, while the bulk FeSO_4_ and FePO_4_ were labeled with ^58^Fe (see “Methods”). We calculated the amounts of ^57^Fe and ^58^Fe labels in blood 14 d after the 2nd and 4th test meals based on the shift in iron-isotopic ratios and the estimated amount of iron circulating in the body^24^. For details of laboratory analyses, calculation of iron absorption and statistical analyses see “Methods”.

Baseline characteristics of the 18 women are shown in Table 1; 14 were iron deficient, 12 had iron deficiency anemia, 15 had normal hemoglobin A (HbA) and three had HbE trait. As shown in Fig. 5b, median (interquartile range [IQR]) fractional iron absorption was 2.87 (1.71–5.02), 7.90 (4.06–11.52), 15.37 (8.63–20.0) and 24.37 (14.64–31.50) from the test meals fortified with bulk FePO_4_, FePO_4_-NP SSA 98 m^2^ g^−1^, FePO_4_-NP SSA 188 m^2^g^−1^, and FeSO_4_, respectively, with significant differences between all groups (for all, p < 0.005). Thus, particle size reduction of FePO_4_ to SSA 98 m^2^ g^−1^ and 188 m^2^g^−1^ increased iron bioavailability 2.8 and 5.4-fold compared to bulk FePO_4_, and achieved a relative bioavailability of 34% and 72% compared to FeSO_4_. In separate linear regressions including C-reactive protein and plasma ferritin (iron status) as independent variables and iron absorption from bulk FePO_4_ and FeSO_4_ combined (standardized β = −0.393, SE = 0.008, p = 0.075) and on iron absorption from the two FePO_4_-NPs combined (standardized β = −0.575, SE = 0.014, p = 0.0027) as outcome variables, plasma ferritin was a stronger negative predictor of iron absorption from the two FePO_4_-NPs (p = 0.007) than from the larger compounds (p = 0.027). Because iron absorption through the usual DMT1 mediated pathway is regulated by iron status^10^, but absorption through simple translocation of nanoparticle iron would likely not be, this is consistent with iron absorption from FePO_4_-NPs being at least partially dependent on the DMT1 mediated pathway.

**Table 1:** Baseline characteristics of the Thai women (n = 18) participating in the iron absorption study.

n	18
Age^a^, years	31.3 ± 7.3
Body weight, kg	51.7 ± 5.3
BMI, kg/m^2^	20.5 ± 1.4
Hemoglobin^b^, g/L	11.5 (9.8–12.4)
Plasma ferritin, µg/L	8.5 (5.0–16.8)
Soluble transferrin receptor, mg/L	7.4 (5.6–10.9)
C-reactive protein, mg/L	1.0 (0.5–2.4)
α-1-acid glycoprotein, g/L	0.49 (0.42–0.52)
Inflammation, n (%)	2 (11)
Anemia, n (%)	14 (78)
Iron deficiency, n (%)	14 (78)
Iron deficiency anemia, n (%)	12 (67)

^a^Mean ± standard deviation (all such values).

^b^Median; interquartile ranges in parentheses (all such values).

## Conclusions

Previous in vitro studies suggested that gastrointestinal uptake of iron NPs could occur through several mechanisms^13,26,27^. Our in vivo data suggest that absorption of FePO_4_-NPs is largely DMT1 dependent (Fig. 3). Supporting our findings, uptake in cell models of the same FePO_4_-NP SSA 188 m^2^g^−1^ used in this study was reduced by DMT1 inhibitors or siRNA targeting of DMT1^14^. However, our findings do not rule out a DMT1 independent route for absorption of NP iron, as our DMT1^int/int^ mice had reduced but still detectable expression of DMT1-exon 1A and DMT1-IRE in the duodenum and this partial knockdown did not affect absorption of the reference compound FeSO_4_ during the experimental period (PND 24–42). We did not determine the expression of other DMT1 isoforms, which could have been responsible for the observed iron absorption, nor measure DMT1 protein levels in duodenal enterocytes to confirm the absence of DMT1 in DMT1^int/int^ knockdown mice. Importantly, our mouse data (Fig. 4) suggest that the biodistribution of FePO_4_-NPs after uptake in anemic DMT1^int/int^ and DMT1^fl/fl^ mice is comparable to FeSO_4_, and there was no abnormal tissue deposition of iron absorbed from FePO_4_-NP in the reticuloendothelial system (spleen, Peyer’s Patches) or in the kidney or liver. In our human study, plasma ferritin was a strong negative predictor of iron absorption from the two FePO_4_-NPs (SSA of 188 m^2^ g^−1^ and 98 m^2^ g^−1^), suggesting physiologic regulation by body iron stores. Taken together, these data argue against, but do not rule out, unregulated translocation and biodistribution of NP iron from gastrointestinal exposure.

Other food-relevant nanoparticles, e.g. SiO_2_, TiO_2_ ZnO and Al_2_O_3_, induce adverse effects in human intestinal cells or experimental animals^28–37^. In contrast, gastrointestinal exposure to a variety of iron NPs has not been associated with measurable toxicity^5,19,26,38–44^. Short-term feeding of iron deficient rats with diets containing iron NPs^5,38^ or iron NPs stabilized on β-lactoglobulin fibrils^43^ did not cause histopathology or oxidative stress. We previously performed extensive toxicity testing of the FePO_4_ NPs used in this study^19^. In rats fed diets containing these iron NPs for 90 days (at doses at which FeSO_4_ has been shown to induce adverse effects), feeding did not cause toxicity, including oxidative stress, organ damage, abnormal tissue iron accumulation or histological changes^19^. Furthermore, they were taken up in vitro by gastrointestinal cells without reducing cell viability or inducing oxidative stress^19^. In these studies, FePO_4_ nanoparticles appeared to be as safe for ingestion as FeSO_4_.

Conflicting information regarding the toxicity of food relevant nanoparticles and regulatory issues have so far hampered applications of nanotechnology in the food sector. However, as described above, in vitro and in vivo studies have consistently shown that iron NPs do not show measurable toxicity on gastrointestinal exposure. Here, we demonstrate that absorption of FePO_4_-NPs is largely via the physiological DMT1 pathway and biodistribution patterns resemble FeSO_4_. In addition, we show that nanostructuring of FePO_4_ sharply increases bioavailability in anemic women and achieves a relative bioavailability of 72% compared to FeSO_4_, confirming previous absorption data in rats^5,38^. Together, these findings suggest the promise of iron NPs as novel food fortificants. However, further research is needed to better clarify the uptake pathways of iron and other mineral NPs from gastrointestinal exposure before they can be recommended for nutritional applications.

## Methods

### Nanoparticle production and characterization

For the production of nanostructured ferric phosphate (FePO_4_-NP) by flame spray pyrolysis (FSP), iron nitrate nonahydrate (purity ≥ 97.0%, Sigma-Aldrich, Buchs, Switzerland) and tributyl phosphate (97%, Sigma-Aldrich) were dissolved in a 1:1 mixture by volume of ethanol (denat. 2% 2-butanone, Alcosuisse) and 2-ethylhexanoic acid (purity ≥ 99%, Sigma-Aldrich) at a total metal concentration of 0.4 mol L^−1^ or 0.5 mol L^−1^ for the two compounds. This precursor solution was fed at 2 or 7 mL min^−1^ into the FSP spray nozzles by a syringe pump (Lambda, VIT-FIT) and atomized by co-flowing 5 or 7 L min^-1^ of oxygen (purity ≥ 99.5%, Pangas) at 1.5 bar pressure drop. The spray was ignited by a methane/oxygen (2.5 L min^−1^) ring-shaped flame^45^. Using a vacuum pump (Busch, Mink MM1202 AV), product particles were collected on water-cooled Teflon membrane-filters (1TMTF700WHT, BHA Technologies AG) placed at least 70 cm above the burner. Pharmaceutical grade (German Pharmacopoeia DAB, Erg. B.6, no. 505033001, Lohmann) amorphous ferric orthophosphate with a SSA of 25 m^2^ g^−1^ served as reference compound for BET and XRD measurements. FePO_4_-NPs for the human study were produced from stable isotope (^57^Fe) enriched precursors, bulk FePO_4_ and FeSO_4_ from (^58^Fe) enriched precursors (Chemgas). SSA was determined by N_2_ adsorption (Micromeritics Tristar 3000, Micromeritics Instruments Corp) at 77 K in the relative pressure range *p*/*p*0 = 0.05–0.25 and calculated using Brunauer–Emmett–Teller (BET) theory. Assuming dense spherical particles, the particle diameter (*d*_BET_) was calculated from the measured SSA according to *d*_BET_ = 6/(ρ·SSA), where ρ is the solid particle density (FePO_4_^*^2H_2_O = 2.87 g cm^−3^)^46^. For transmission electron microscopy (TEM) analysis, the powders were deposited on a parlodion foil supported on a copper grid and analyzed on a CM12 microscope (FEI, LaB6 cathode, operated at an acceleration voltage of 100 kV). The crystallinity of the powders was investigated by X-ray diffraction (XRD) on a AXS D8 Advance diffractometer (Bruker) operating with a Cu–Kα radiation. Hydrodynamic diameter was determined by dynamic light scattering using a Zetasizer Nano ZS (Malvern).

### Animal studies

#### Intestine-specific DMT1 knockdown (DMT1^int/int^) model

Intestine-specific DMT1 knockdown (DMT1^int/int^) mice were bred by crossing floxed DMT1 (DMT^fl/fl^) mice on a homogenous C57BL/6 strain (courtesy of Nancy Andrews, Duke University, USA^21^) with villin-Cre transgenic mice on the C57BL/S6J strain (ETH Zurich). The floxed DMT1 mice were back-crossed to a C57BL/6J background for six generations to establish the DMT1^fl/fl^ mouse breeding colony at ETH Zurich. The 6th generation was then shipped to the Vivarium at NWU to establish the intestine-specific DMT1 knockdown and villin-Cre mouse breeding colony for the experiments described in this article. To obtain DMT1^int/int^ mice, the animals needed to be homozygously floxed and have the villin-Cre transgene (Cre-positive). The homozygously floxed littermates (DMT^fl/fl^) that did not have the villin-Cre transgene (Cre-negative) served as control in all mice experiments to account for a potential effect of floxing on iron absorption and biodistribution. To breed these mice, we mated heterozygously floxed males that have the villin-Cre (Cre-positive) transgene with homozygoulsy floxed females without the villin-Cre transgene (Cre-negative). Theoretically, this results in an approximate yield of 25% DMT1 KO and 25% controls per litter. In order to obtain the required number of mice for the different experiments, we continuously bred, genotyped and enrolled mice into the experiments. Thus, mice in the different experimental groups are from different litters.

#### Genotyping

The genotyping method to screen each mouse bred from the floxed DMT1 and villin-Cre mice colony was as follows. Briefly, a tissue sample (distal tail sample [≤ 2 mm]) was collected at PND 10–21^47^. gDNA isolation was done using the GenElute™ Mammalian Genomic DNA (gDNA) Miniprep Kit (Sigma Aldrich) following the manufacturers protocol. The quality of DNA was assessed and quantified using the NanoDrop™ spectrophotometer (ND-1000, Wilmington, DE, USA). Gene specific polymerase chain reaction (PCR) was performed and amplicons were visualized using ethidium bromide stained gel electrophoresis. For PCR amplification of the DMT1 gene the forward primer 5’-atgggcgagttagaggcttt-3’ and the reverse primer 5’-cctgcatgtcagaaccaatg-3’ were used. For PCR amplification of the villin-Cre gene the forward primer 5’-gtgtgggacagagaacaaacc-3’ and reverse primer 5’-acatcttcaggttctgcggg-3’ were used together with an endogenous control (MyD88) primer pair 5’-agacaggctgagtgcaaacttgtgctg-3’ and 5’-ccggcaactagaacagacagactatcg-3’. Control gDNA with known genotype (ETH) as well as none-template control samples were included in each PCR run.

#### Housing and diets

Mice (for breeding and in experiments) were housed in polysulfone individually ventilated cages (391 × 199 × 160 mm [WxDxH]) (Tecniplast, UK) with Alpha-Dri® alpha cellulose bedding (Alpha-Dri, Shepherd Speciality Papers) (< 2.00 ppm iron) under a 12/12 h light/dark cycle (lights on at 06:00) at 22 ± 2 °C and 55 ± 10% relative humidity. The diets used in the experiments and for breeding of experimental mice were commercially obtained purified diets according to AIN93-G standard^48^, with modifications in iron content and compound. Iron fortified diets contained 35 mg iron per kg (ppm) diet, while iron deficient reference diets contained 3 ppm iron (native iron only). The diets were produced by Dyets Inc. (2508 Easton Avenue, P. O. Box 3485, Bethlehem, PA 18017, USA). Iron content of diets was analyzed in spot samples from each batch by Covance Laboratory Services (Madison, WI, USA) before shipping. All mice had ad libitum access to food and to deionized water (18 mΩ). Mice were weighed three times per week (or more frequently during experiments) to monitor weight gain.

#### Ethics

All animal experiments were approved by the Animal Ethics Committee of the Faculty of Health Sciences of the North-West University (NWU-00050-16-A5 & NWU-00258-17-A5), Potchefstroom, South Africa, and were conducted following the 3R principles for animal research and the Animals in Research: Reporting In Vivo Experiments (ARRIVE) guidelines^49^.

#### Characterization of intestine-specific DMT1 knockdown model

A total of 10 DMT1^int/int^ (n = 6 male; n = 4 female) and 21 DMT1^fl/fl^ (n = 10 male, n = 11 female) mice, born to dams that were kept on the purified AIN-93G diet containing 35 ppm iron (as ferrous citrate) ad libitum, were randomly allocated in pairs to receive an iron deficient (3 ppm native iron) or iron-sufficient (35 ppm ferrous citrate) diet ad libitum from PND 24 to PND 42. PND 42 was set as endpoint, as this was the time point when first mice reached an Hb < 4 g dL^−1^, which we defined as humane endpoint^22^ (a humane endpoint is the earliest scientifically justified point at which pain or distress in an experimental animal can be prevented, terminated, or relieved, while meeting the scientific aims and objectives of the study^50^). Hemoglobin (Hb) concentrations were measured in tail blood spots (20 µg dL^−1^) at PND 24, 27, 30, 33, 36, 39 and 42 using a calibrated Hb 201 + HemoCue® system (HemoCue Angelholm, Sweden). At PND 42, mice were euthanized by decapitation, and liver, duodenum and colon tissue immediately removed, snap frozen in liquid nitrogen, and stored at −80 °C until analysis.

Expression of DMT1 mRNA harboring an iron-responsive element (IRE) in its 3’-terminal exon and the upstream 5’ exon1A in duodenum, colon and liver was analyzed with qPCR. PCR ready Syber green primers were synthesized by IDT (WhiteHead Scientific, South Africa). Primer pair sequences were adapted from Hubert and Hentze (2002)^23^. Total RNA was isolated by using Trizol reagent following the standarized protocol. cDNA synthesis was performed using 10 mM oligo-dT_18mer_ and random hexamer primer mix with Superscript II reverse transcriptase (Qiagen), following the method prescribed by the manufacturer. A total of 50 ng cDNA was used as template in triplicate qPCR reactions with QuantiNova sybr green 2 × master mix together with 1 mM syber green ready primers. Reactions were prepared and amplification was performed at 94˚C for 20 s, 55 ˚C at 40 s and 72 ˚C at 30 s for 30 cycles. Gene expression was deduced using 18S and βActin as endogenous reference genes to calculate delta Ct values for further statistical analysis.

#### Eighteen-day FePO_4_-NP feeding study in DMT1^int/int^ and DMT1^fl/fl^ mice

A total of 15 DMT1^int/int^ (n = 6 male; n = 9 female) and 13 DMT1^fl/fl^ (n = 6 male, n = 7 female) mice, born to dams that were kept on the standardized AIN-93 G diet containing 35 ppm iron (as ferrous sulphate [FeSO_4_]) ad libitum, were randomly allocated in pairs to receive a diet fortified with 35 ppm FePO_4_-NP (SSA 98 m^2^g^−1^) or FeSO_4_ (reference compound) added to an iron-free AIN93-G diet from PND 24 to PND 42 ad libitum. Hb concentrations were measured in tail blood spots at PND 24, 27, 30, 33, 36 and 42 using the Hb 201 + HemoCue® system (HemoCue Angelholm, Sweden). At PND 42, mice were euthanized by decapitation and liver tissue immediately removed, snap frozen in liquid nitrogen, and stored at −80 °C until analysis.

Liver tissue samples were homogenized and digested with nitric acid according to Erikson et al. (1997)^51^, and total iron concentrations were measured by using the hydrogen reaction mode on an Agilent 7900 quadrupole ICP-MS at the Central Analytical Facilities, Stellenbosch University, South Africa. Samples were introduced via a 0.4 ml/min micromist nebulizer into a peltier-cooled spray chamber at a temperature of 2 °C. The instrument was optimized for analysis in high matrix introduction (HMI) mode, and all samples and standards were diluted with argon gas to minimize matrix load to the analyzer. The instrument was calibrated using a National Institute of Standards and Technology (NIST) traceable standard (Inorganic Ventures, USA). NIST-traceable quality control standards at high and low concentration levels (De Bruyn Spectroscopic Solutions, Bryanston, South Africa) were analyzed to verify the accuracy of the calibration before sample analysis commenced and this was repeated for every 12 samples to monitor drift. A germanium (Ge) internal standard was introduced online to monitor instrument drift and correct for matrix differences between samples and standards. During the course of the analysis, internal standard recovery was between 90 and 110% for all samples, and recovery for drift monitor standards between 95 and 105%. Oxide formation was less than 0.3%. Three replicate measurements were completed for each sample.

#### Absorption and biodistribution of radiolabeled FePO_4_-NP and FeSO_4_ from an acute oral dose in iron deficient anemic DMT1^int/int^ and DMT1^fl/fl^ mice

A total of 13 DMT1^int/int^ (n = 7 male; n = 5 female) and 15 DMT1^fl/fl^ (n = 7 male, n = 8 female) mice, born to dams that were kept on the standardized AIN-93 G diet containing 35 ppm iron (as FeSO_4_) ad libitum, were placed on an iron deficient diet (3 ppm native iron) from PND 21 and throughout the entire experiment. Mice were randomly allocated to receive radiolabeled FePO_4_-NP (SSA 98 m^2^g^−1^) or FeSO_4_. At PND 24, mice were transported to the South African Nuclear Energy Corporation South Africa (NECSA) and left to acclimatize to the new environment until administration of radiolabeled FePO_4_-NP or FeSO_4_ by oral gavage at PND 30 (29–31). The day before compound administration, Hb was measured in a tail blood spot.

Mice were fasted for 2 h before administration of the oral gavage during which they were acclimatized to metabolic cages (3701M081; Tecniplast). Then, a single dose of ~ 50 µg iron in the form of the allocated compound labelled with ^59^Fe (mean activity: 0.30 MBq) was orally gavaged (in 100 µL saline containing 0.1% bovine serum albumin) using disposable flexible gavage needles. Total dose administered was determined by measuring syringe activity before and after gavage using a dose calibrator (Capintec CRC-15R, Capintec Inc., Ramsey, NJ, USA). Mice were then placed into a clean metabolic cage and received ad libitum access to iron deficient diet (3 ppm iron) one hour after oral gavage. After 24 h, the mice were euthanized by decapitation and the following individual organs were dissected and analyzed for ^59^Fe content and weighed using an automated Hidex®600 SL gamma-counter (Hidex Oy, Finland). All counts were adjusted for decay: whole blood, heart, lung, liver, spleen, stomach, duodenum, peyer’s patches, ileum, jejenum, colon, kidneys, femur, as well as feces and urine. Chyme was separated from the intestinal segments by washing with 1% phosphate-buffered saline.

A total of 15 mg of the nanostructured FePO_4_ (SSA 98 m^2^g^-1^) was irradiated in the SAFARI-1 20 MW research reactor in a hydraulic position at a neutron flux of 1 × 10^14^ n/cm^2^s for 16 days with a 3 day cooling period. Irradiation provided 512 MBq ^59^Fe (t_½_ = 44.5 days) per 1 mg of FePO_4_-NPs^52^. The color of the FePO_4_-NP (SSA 98 m^2^ g^−1^) remained yellowish during the irradiation given confidence that the particles retained their nano structure. This was further confirmed by measuring the surface area using a Tristar 3000 BET surface area and porosity analyzer (Micromeretrics, Norcross, USA). Using a special small volume adapter the SSA of the irradiated particles was determined to be 79.1 m^2^ g^−1^ which mimicked the SSA determined prior to irradiation. Special care was taken to remove the static nature of the NP after irradiation using a Antistatic Ionizer (RADAWG, Radom, Poland), before opening and during handling of the radioactive labelled NPs. FePO_4_-NP were decayed a further 30 days prior to animal administration to allow for the co-activated ^32^P (t_½_ = 14 days) to decay. ^32^P is a pure β emitter and hence did not interfere with the determination using gamma spectrometry at the > 1000 keV range^52^. ^59^Fe-labelled FeSO_4_ was purchased from American Radiolabeled Chemicals, Inc. (St. Louis, USA) with a specific activity > 185 GBq/g. In order to mimic the chemical administrated dose used for the NP’s (50 µg and 0.3 MBq), non radioactive FeSO_4_ was added to a subset of the purchased stock solution. ^59^Fe radioactivity (for administration) was detected by a dose calibrator (Capintec CRC-15R, Capintec Inc., Ramsey, NJ, USA), while samples were analyzed using an automated Hidex^®^600 SL gamma-counter (Hidex Oy, Finland) making use of the 1099 keV 56.5% gamma emission. Cross calibration between the two instruments were obtained by using a ^59^Fe standard curve after a series of dilutions. The irradiated particles were dispersed in in saline containing 0.1% bovine serum albumin by mixing for 30 s on a vortex followed by sonication for 10 min in a Sonorex Digitec waterbath (Bandelin Electronic) at 35 kHz and 80 W and were gavaged within 15 min after sonication. We acknowledge that we did not determine absorption and biodistribution of FePO_4_-NP with an SSA of 188 m^2^ g^−1^ in our mouse experiments because we were not able to radio-label this compound without colour and structural changes (visual inspection) after irradiation in the reactor as opposed to the 98 m^2^ g^−1^ where this was not observed.

#### Statistical analysis

Data were analyzed using IBM SPSS Statistics software (version 24). Data were examined for normality of distribution (using q–q plots, histograms, and Shapiro–Wilk test) and the presence of outliers (using box plots). Homogeneity of variance was examined by the Levene’s test. Variables that significantly deviated from normality and/or variance of homogeneity were transformed prior to interferential statistical analysis. Differences in Hb trajectories over time (PND 24–42) by genotype (DMT1^int/int^ vs. DMT1^fl/fl^) and by dietary iron content (35 ppm vs. 3 ppm) or iron compound (FePO_4_-NT vs. FeSO_4_) were determined using repeated measures ANOVA. Differences in tissue iron concentrations, gene expression and percentage initial ^59^Fe dose/g tissue by genotype and by iron compound were determined by two-sided independent t-tests. The results were expressed as means ± SEM and differences were considered significant at p < 0.05.

### Human study

#### Design and subjects

In a randomized cross-over study, iron deficient, mostly anemic Thai women (n = 18) aged 18 to 49 years consumed four test meals containing ^57^Fe-labeled FePO_4_-NP (SSA 98 m^2^g^−1^), ^57^Fe-labeled FePO_4_-NP (SSA 188 m^2^g^−1^), ^58^Fe-labeled bulk FePO_4_ (negative reference compound) and ^58^Fe-labeled FeSO_4_ (positive reference compound) in random order (using a computerized random number generator—Excel). Stable iron isotope incorporation in red blood cells was determined 14 days after test meal administration.

Women were eligible to participate in the study if: (1) female aged 18 to 49 years; (2) body mass index (BMI) < 23 kg/m^2^ and body weight < 65 kg; (3) Hb ≥ 80 g/L and plasma ferritin < 25 µg/L; (4) not pregnant (confirmed by pregnancy test) or lactating; (5) healthy, no chronic diseases or medications (except oral contraceptives) and no inflammation (C-reactive protein (CRP) < 5 mg/L); (6) no blood donation or significant blood loss at least 4 months before study start; (7) no consumption of vitamin or mineral supplements at least 2 weeks before study start; (8) normal hemoglobin A (HbA) or HbE trait; and (9) nonsmokers.

Sample size calculations indicated that 16 women should be included based on 80% power to detect a 40% difference in iron bioavailability within subjects, an SD of 8.2% for log-transformed absorption data from previous absorption studies with the same meal and iron source/compound in a similar population of Thai women, and a type I error rate of 5%. We anticipated a dropout rate of 10% and therefore recruited 18 women.

#### Ethics

The study has been carried out according to the guidelines laid down in the Declaration of Helsinki, and all procedures involving human study participants were approved by the Ethics Committee of ETH Zurich (Zurich, Switzerland) and the Mahidol University Central Institutional Review Board (Salaya, Nakhon Pathom, Thailand). The study was registered at clinicaltrials.gov as NCT03660462 on 06/11/2018. Detailed oral and written information explaining the study purposes and potential risks and benefits were provided to the interested volunteers. Written informed consent was obtained from all participants. The oral administration of stable isotopes does not present any health risk. All data were coded and treated confidentially.

#### Study procedures

Women were recruited by screening at the Institute of Nutrition at Mahidol University and at the Khlong Yong Health Promoting Hospital in Nakhon Pathom, Thailand. All details of the study were explained to them, and if they were interested in participating in the study, they were asked to sign the written informed consent form. Then, weight and height were measured (to calculate BMI), and a venous blood sample collected to determine Hb, serum ferritin, and C-reactive protein concentrations. Women who fulfilled all the inclusion criteria were instructed to not eat red meat, fish or poultry four days prior to the scheduled test meals.

In a first phase, the women consumed two randomly assigned test meals (A, B, C or D) between 7.00 and 9.00 am after an overnight fast on study day 1 and 2. In the second phase, the women consumed the remaining randomly assigned test meals (A, B, C or D) between 7.00 and 9.00 am after an overnight fast on day 16 or 17. To distinguish between the absorption of the two forms of ^57^Fe-labeled FePO_4_-NP (SSA 98 m^2^g^−1^ and SSA 188 m^2^g^−1^) and between ^58^Fe-labeled FeSO_4_ and FePO_4_, the subjects consumed one Fe compound labeled with ^57^Fe and one with ^58^Fe in each phase. The test meals (see details below) were fortified with 2 mg of the respective isotopically labelled iron compound and administered with a glass of deionized water (200 ml). Fourteen days after the last teast meal administration in the first phase (day 16) and again fourteen days after the last test meal administration in the second phase (day 31), a venous blood sample was taken to determine iron absorption.

#### Composition of the test meal

The test meal was composed of steamed white rice (50 g dry weight), which was served with a vegetable soup prepared from local vegetables (50 g white cabbage, 50 g Chinese cabbage, 30 g Thai mushrooms and 20 g steamed carrots) in 120 mL of water. All ingredients were purchased in bulk and used for the entire study. The food portions were kept frozen until use, and each portion was microwaved on the day of feeding.

#### Stable-isotope labels

^58^FeSO_4_ were prepared from ^58^Fe-enriched elemental iron (> 99.8% isotopic enrichment; Chemgas) by dissolution in 0.1 mol/L sulfuric acid. ^58^FePO_4_ was prepared from ^58^Fe-enriched elemental iron (> 99.8% isotopic enrichment; Chemgas). The two FePO_4_-NP (SSA 98 m^2^g^−1^ and 188 m^2^g^−1^) were prepared from ^57^Fe-enriched elemental iron (> 99.8% isotopic enrichment; Chemgas) by flame spray pyrolysis as described previously^5^. We analyzed the labeled iron compounds for iron isotopic composition and the tracer iron concentration via isotope-dilution mass spectrometry as described below.

### Laboratory analyses

Venous blood samples were drawn into EDTA-treated tubes. We measured Hb immediately after blood draw with the use of hematology analyzer (Sysmex, Kobe, Japan) and quality controls provided by the manufacturer before each assessment. Hemoglobin typing for β-globin abnormality was done by using HPLC (Variant Hemoglobin Testing System; BioRad, Hercules. CA) with calibrators and controls provided by the manufacturer. DNA analysis for α-globin abnormalities was done by using a GeneAmp PCR System (Applied Biosystem, Foster City, CA) and a Gel Doc 2000 Gel Documentation System (BioRad, Hercules, CA). A 500 µL aliquot of whole blood was frozen for isotopic analysis (see below). The remaining blood was centrifuged and the plasma aliquoted and stored at − 20 °C. Whole blood and plasma were shipped frozen to the ETH Zurich, Switzerland, for analysis of iron (plasma ferritin and soluble transferrin receptor) and inflammation (C-reactive protein, alpha-1-acid glycoprotein) parameters using a multiplex immunoassay^53^. Anemia was defined as Hb < 120 g/L^54^. Iron deficiency was defined as plasma ferritin < 12 mg/L and/or soluble transferrin receptor > 8.3 µg/mL^53^, and iron deficiency anemia was defined as Hb < 120 g/L^54^ and plasma ferritin < 12 mg/L and/or soluble transferrin receptor concentration > 8.3 µg/ml^53^. Normal C-reactive protein and alpha-1-acid glycoprotein concentrations for this assay in healthy adults are < 5 mg/L and < 1 g/L, respectively^53^.

#### Calculation of iron absorption

Whole blood samples were mineralized in duplicate with the use of a nitric acid and microwave digestion followed by separation of the iron from the blood matrix via anion-exchange chromatography and a subsequent precipitation step with ammonium hydroxide. We measured iron isotope ratios by using an inductively coupled plasma mass spectrometer (Neptune, Thermo Finnigan, Germany) equipped with a multicollector system for simultaneous iron beam detection^55^. We calculated the amounts of ^57^Fe and ^58^Fe isotopic labels in blood 14 days after administration of the test meals based on the shift in iron-isotopic ratios and the estimated amount of iron circulating in the body. We remeasured the baseline isotopic composition in blood at the start of the second phase of test meals. Circulating iron was calculated based on the blood volume that was estimated from body length and weight at endpoint measurement according to Linderkamp et al.^56^ and measured Hb (mean Hb from baseline and endpoint). The calculations were based on the methods described by Turnlund et al.^57^ and Cercamondi et al.^58^, taking into account that iron isotopic labels are not monoisotopic.

#### Data and statistical analysis

Data were analyzed using SPSS (IBM SPSS statistics, version 22.0). Normally distributed data were presented as mean ± SD, and not normally distributed data as median (IQR). Repeated-measures ANOVA was used to assess the effect of iron compound on square root-transformed fractional iron absorption. Fractional iron absorption was the dependent variable and the iron compound was added to the model as the independent variable; pairwise comparisons were performed using two-sided paired t-tests with Bonferroni adjustment for multiple testing. Separate linear regressions were done to compare predictors of iron absorption from the nano-sized iron compounds and the bulk sized compounds. C-reactive protein and serum ferritin (iron status) were added as independent variables with the dependent variable being (1) iron absorption from bulk FePO_4_ and FeSO_4_ and (2) iron absorption from the two FePO_4_-NPs. Significance was set at *P* < 0.05.

## Supplementary Information

Supplementary Figure S1.

Supplementary Figure S2.

## Data Availability

All relevant data are included in the manuscript. These are also available by the authors upon request.
