# Supplementary material for: Iron from nanostructured ferric phosphate: absorption and biodistribution in mice and bioavailability in iron deficient anemic women
Source: Sci Rep. 2022 Feb 18;12:2792. doi: 10.1038/s41598-022-06701-x (PMC8857185; doi:10.1038/s41598-022-06701-x)
Supplement: Supplementary file 1 — Supplementary Figure S1. [file 41598_2022_6701_MOESM1_ESM.pdf]

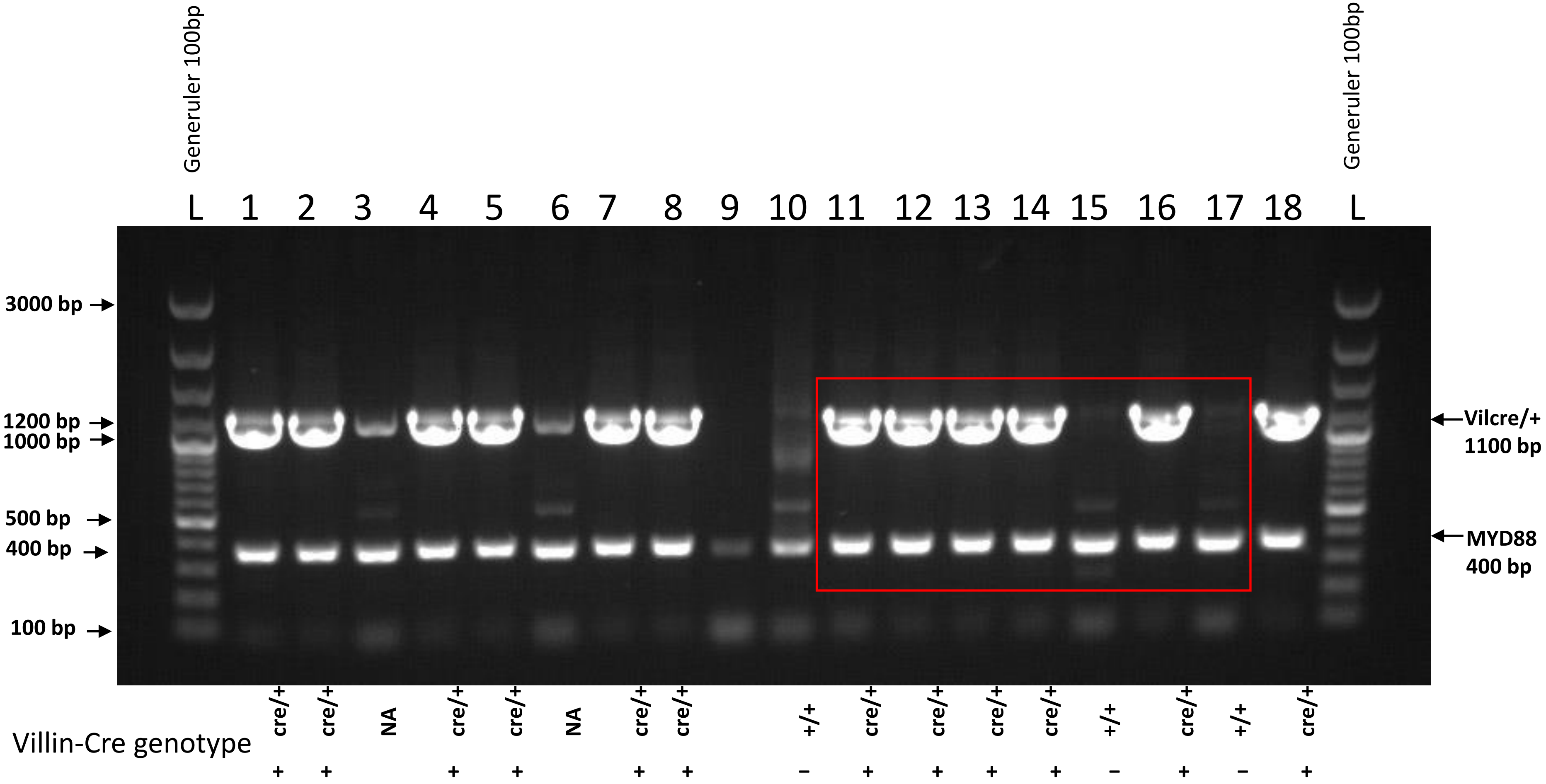

Supplementary Figure 1: Villin-cre genotyping visualised with a 1 % TAE Ethidium bromide stained agarose gel. Generuler 100 bp used as DNA marker. Electrophoresis was done for 30 min at 120V. Red squared section cropped and compiled as Figure 2a.
